# Endoplasmic reticulum stress in steroid-induced osteonecrosis: translational bottlenecks and targeted therapeutic strategies

**DOI:** 10.3389/fphar.2026.1856325

**Published:** 2026-07-02

**Authors:** Tianpeng Liu, Yi Shen, Wenhao Wang, Furui Fu, Mengting Yuan, Jin Huang, Hongbin Xu, Yong He, Haifeng Jia, Jie Wang, Xin Wei, Haitao Zhang, Xiang Gao, Dezhi Tang

**Affiliations:** 1 Longhua Hospital, Shanghai University of Traditional Chinese Medicine, Shanghai, China; 2 Institute of Spine, Shanghai University of Traditional Chinese Medicine, Shanghai, China; 3 Graduate School, Shanghai University of Traditional Chinese Medicine, Shanghai, China; 4 Key Laboratory of Theory and Therapy of Muscles and Bones, Ministry of Education, Shanghai, China; 5 Huadong Hospital Affiliated to Fudan University, Shanghai, China; 6 The First Affiliated Hospital of Guangzhou University of Chinese Medicine, Guangzhou, China

**Keywords:** endoplasmic reticulum stress, exosomes, HIF-1α, steroid-induced osteonecrosis of the femoral head, targeted drug delivery

## Abstract

Steroid-induced osteonecrosis of the femoral head (SONFH) is a crippling orthopedic disease. While glucocorticoids are a well-established etiological factor, the molecular framework driving the cascading tissue destruction remains elusive. Endoplasmic reticulum stress (ERS) has emerged as a central pathogenic hub. This review comprehensively synthesizes current evidence on how ERS orchestrates the multi-dimensional pathological network in SONFH, focusing on specific signaling paradoxes and translational barriers. Mechanistically, ERS drives irreversible osteoblast apoptosis via the PERK-CHOP axis and impairs endothelial regeneration by suppressing mTOR signaling. Notably, ERS exhibits profound microenvironmental heterogeneity: in articular chondrocytes, it aberrantly stabilizes HIF-1α to precipitate matrix degradation—a stark contrast to its canonical vasculoprotective role. Furthermore, ERS exacerbates intraosseous ischemia through a positive feedback loop with lipid metabolism. Importantly, we identify the “ischemic island” effect (a physical sclerotic barrier) and inherent biomechanical discrepancies in quadrupedal animal models as primary bottlenecks hindering the clinical translation of potent ERS inhibitors. To bridge this translational chasm, we propose combinatorial interventions coupling ERS modulators with advanced bone-targeted delivery systems (e.g., Asp8-modified nanocarriers) to breach the ischemic barrier, offering a pivotal strategy for future precision joint-preserving therapies.

## 1 Introduction

Osteonecrosis of the femoral head (ONFH) constitutes a highly disabling osteoarticular disease, with steroid-induced osteonecrosis representing the predominant etiological factor among non-traumatic cases, accounting for 30%–50% following high-dose or prolonged glucocorticoid (GC) administration (Weinstein, 2011; Ikeuchi et al., 2015; Cui et al., 2016). Once the disease progresses to subchondral bone collapse, total hip arthroplasty often becomes inevitable (Birla et al., 2021). Previous pathogenic hypotheses, including “vascular thrombosis,” “lipid metabolism dysregulation,” and “intraosseous hypertension,” have partially explained certain pathological features (Zhang et al., 2024; Zheng et al., 2025), however, these theories often operate in isolation, failing to elucidate how GCs, as a single insult, simultaneously trigger the complex cascading response encompassing vascular injury, osteoblast death, and metabolic reprogramming (Chen et al., 2025). Identifying an upstream mechanism capable of integrating these multi-dimensional pathological alterations represents the critical step toward understanding disease pathogenesis.

ERS functions as a sentinel for cells to perceive microenvironmental perturbations such as hypoxia and calcium imbalance, and has emerged as a pivotal candidate for unraveling this enigma (Tabas and Ron, 2011). When GC overload drives the accumulation of unfolded proteins beyond a critical threshold, the UPR undergoes a “qualitative switch” from adaptive protection to activation of lethal factors such as C/EBP homologous protein (CHOP), thereby initiating cell death programs (Tabas and Ron, 2011). However, interpreting ERS function solely through the apoptosis lens remains reductionistic. Accumulating evidence demonstrates that ERS essentially operates as a multifunctional signaling hub that orchestrates angiogenesis via the mechanistic target of rapamycin (mTOR) pathway and actively participates in lipid metabolism reprogramming (Celik et al., 2023).

Notably, existing investigations frequently overlook the heterogeneity of signaling pathways across distinct cellular subpopulations—for instance, the same hypoxia-inducible factor may yield diametrically opposite outcomes in endothelial cells versus chondrocytes. Moreover, despite promising experimental efficacy of various ERS-targeting agents, few have successfully achieved clinical translation, highlighting the urgent need to systematically elucidate tissue-specific discrepancies and drug delivery barriers.

This review aims to provide a panoramic overview of the pathogenic mechanisms whereby ERS drives the “ischemia-necrosis-metabolic dysregulation” network in SONFH, with particular emphasis on dissecting the mechanistic paradoxes surrounding molecules such as hypoxia-inducible factor-1α (HIF-1α). We anticipate that this integrated framework will furnish a theoretical foundation for developing precision joint-preserving therapies.

## 2 Molecular mechanisms of endoplasmic reticulum stress

The endoplasmic reticulum (ER) serves not merely as the principal organelle for protein folding and modification in eukaryotic cells, but also as the pivotal calcium reservoir maintaining intracellular calcium homeostasis (Hetz and Papa, 2018; Xian et al., 2024). Upon exposure to external insults such as high-dose glucocorticoids, ischemia-hypoxia, or oxidative stress, the ER lumen aberrantly accumulates misfolded or unfolded proteins—a state of homeostatic imbalance termed ERS (Liu et al., 2022; Ichiseki et al., 2006; Remondelli and Renna, 2017). To re-establish internal homeostasis, cells initiate the UPR, orchestrated by three ER transmembrane sensor proteins: protein kinase R-like ER kinase (PERK), inositol-requiring enzyme 1 (IRE1), and activating transcription factor 6 (ATF6) (Chen and Cubillos-Ruiz, 2021).

In the quiescent state, these three sensors remain inactive through association with the chaperone glucose-regulated protein 78 (GRP78, also known as binding immunoglobulin protein/BiP) (Li and Lee, 2006); Upon stress stimulation, GRP78 dissociates to bind unfolded proteins, thereby liberating the sensors to initiate downstream cascades. ①The PERK pathway: Serving as a critical regulator of protein synthesis, activated PERK rapidly attenuates global protein translation by phosphorylating eukaryotic initiation factor 2α (eIF2α), thereby alleviating the ER folding burden (Harding et al., 2000; Ron, 2002). However, if stress intensity surpasses a critical threshold, persistently phosphorylated eIF2α selectively induces the expression of activating transcription factor 4 (ATF4), which subsequently activates the core pro-apoptotic factor CHOP, driving the cellular switch from survival mode to death execution (Zinszner et al., 1998). ②The IRE1 pathway: IRE1α represents the evolutionarily most conserved sensor, endowed with both kinase and endoribonuclease activities. It generates transcriptionally active XBP1s by splicing X-box binding protein 1 (XBP1) mRNA, thereby upregulating chaperone proteins and lipid synthesis-related genes (Yoshida et al., 2001). Additionally, hyperactivated IRE1 can recruit tumor necrosis factor receptor-associated factor 2 (TRAF2) and activate c-Jun N-terminal kinase (JNK), thereby mediating inflammatory responses and apoptotic signaling (Urano et al., 2000). ③The ATF6 pathway: Upon sensing ER stress, ATF6 translocates to the Golgi apparatus (Haze et al., 1999), where proteolytic processing by site-1 protease (S1P) and site-2 protease (S2P) liberates its active fragment for nuclear entry (Ye et al., 2000). This cleaved fragment functions as a transcription factor that potently induces chaperone proteins such as GRP78, augmenting ER folding capacity.

This intricate signaling network exhibits a profound dualistic effect: early UPR endeavors to restore homeostasis by augmenting folding capacity (pro-survival), whereas persistent and robust UPR ultimately activates lethal signals including CHOP and JNK (pro-apoptotic). This “adaptive-to-apoptotic” switch constitutes the molecular basis for tissue necrosis in SONFH (Sano and Reed, 2013; Liang et al., 2020).

## 3 ERS-mediated pathogenic mechanisms in ONFH

### 3.1 ERS-driven osteoblast and osteocyte apoptosis

**FIGURE 1:**
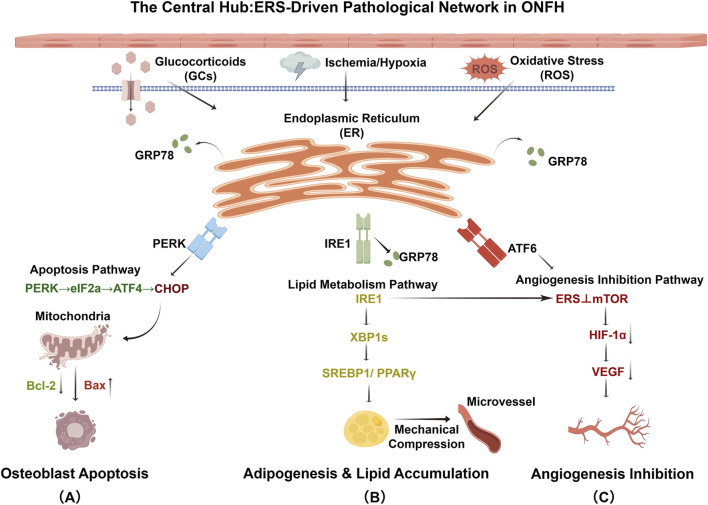
ERS as the central hub in ONFH pathogenesis. Glucocorticoids (GCs) and ischemia activate the unfolded protein response (UPR) via three transmembrane sensors: PERK, IRE1, and ATF6. **(A)** Apoptotic axis: The PERK-eIF2α-ATF4 signaling pathway activates the pro-apoptotic factor CHOP, leading to mitochondrial dysfunction (Bcl-2↓/Bax↑) and osteoblast apoptosis. **(B)** Metabolic axis: The IRE1-XBP1 pathway upregulates SREBP1 and PPARγ, promoting adipogenic differentiation and lipid accumulation, thereby mechanically compressing microvessels. **(C)** Angiogenic axis: ERS inhibits the mTOR pathway, downregulating HIF-1α and VEGF expression, thereby impairing angiogenesis and exacerbating ischemia (Created with Figdraw). Abbreviations: CHOP, C/EBP homologous protein; SREBP1, sterol regulatory element-binding protein 1; PPARγ, peroxisome proliferator-activated receptor γ; HIF-1α, hypoxia-inducible factor-1α; VEGF, vascular endothelial growth factor.

The survival of osteocytes and osteoblasts constitutes the cornerstone for maintaining trabecular integrity within the femoral head (Kuang et al., 2019). Accumulating evidence demonstrates that GC-induced ERS potentiates the apoptotic cascade by functioning as a critical upstream initiator (Guo et al., 2021). Specifically, glucocorticoids dose-dependently activate the PERK-ATF4-CHOP signaling axis, wherein CHOP operates as a core pro-apoptotic transcription factor that simultaneously downregulates anti-apoptotic Bcl-2 while upregulating pro-apoptotic Bim (Bcl-2-like protein 11) and Bax (Bcl-2-associated X protein), thereby precipitating mitochondrial dysfunction (López-Royuela et al., 2010). Furthermore, the functional crosstalk between the ER and mitochondria is crucial for executing this apoptotic program, largely mediated by mitochondria-associated ER membranes (MAMs). During prolonged GCs exposure, structural tethering at MAMs facilitates the excessive transfer of calcium ions (Ca^2+^) from the ER to the mitochondria via the IP3R-GRP75-VDAC1 complex (Figure 1A). This severe calcium overload induces mitochondrial membrane depolarization, accelerating the release of cytochrome c and committing the osteoblasts to irreversible apoptosis (De Ridder et al., 2023; Szabadkai et al., 2006).

Notably, dopamine receptor D1 (DRD1) has been shown to modulate this axis via the cAMP/PKA pathway, influencing osteoblast survival (Zheng et al., 2025). Apart from the classical mitochondrial pathway, Caspase-12—an ERS-specific apoptosis initiator—is significantly activated in SONFH models, directly cleaving Caspase-9 and amplifying Caspase-3 signaling to constitute a mitochondria-independent secondary death pathway (Guo et al., 2021; Yang et al., 2017).

### 3.2 Vascular endothelial injury and angiogenesis suppression

Impaired microcirculation within the femoral head is widely recognized as the initiating insult of necrosis, whereas vascular endothelial cells (ECs) exhibit high sensitivity to ERS (Yao et al., 2020). High-dose GC exposure significantly elevates GRP78 and CHOP levels in ECs, wherein hyperactivation of the PERK pathway constitutes the core mechanism mediating endothelial apoptosis (Gao et al., 2020). Notably, PERK inhibition (e.g., GSK2656157) significantly improves femoral head blood supply (Gao et al., 2020). Beyond direct induction of apoptosis, ERS impairs endothelial paracrine function by inhibiting mTOR signaling (Chen et al., 2022). This inhibition downregulates downstream HIF-1α and vascular endothelial growth factor (VEGF) expression, directly hindering angiogenesis (Tang et al., 2025). This mechanism explains why necrotic regions fail to establish effective collateral circulation, thereby forming a vicious cycle of “ischemia-induced ERS, ERS exacerbating ischemia” (Figures 1B,C).

### 3.3 Signaling interactions and mechanistic paradoxes

ERS action in SONFH cannot be viewed in isolation; rather, it functions as a multifunctional signaling hub that mediates complex interactions with autophagy, energy metabolism, and lipid synthesis pathways, exhibiting significant heterogeneity across distinct cellular microenvironments.

#### 3.3.1 Dynamic crosstalk exists between ERS and autophagy

In the context of SONFH, this ERS-autophagy crosstalk primarily impacts both osteoblasts and bone marrow microvascular endothelial cells. During the early stress phase, ERS induces protective autophagy to clear unfolded proteins. The UPR signaling upregulates initiation and elongation factors such as Beclin-1, ATG7, and LC3-II to promote phagophore maturation (Sheng et al., 2012); however, under persistent pathological stimuli characteristic of SONFH, this equilibrium collapses. ATG5 (autophagy-related 5) emerges as a pivotal mediator that orchestrates the autophagic switch from pro-survival to pro-apoptotic by interacting with cell death pathways (Wang et al., 2021). Furthermore, prolonged glucocorticoid exposure not only downregulates these initiation factors but also severely impairs the later stages of autophagy, specifically blocking autophagosome-lysosome fusion. This profound blockade in autophagic flux leads to the toxic accumulation of autophagosomes, which aggressively exacerbates ER stress (Li et al., 2018). Consequently, this vicious cycle abrogates the self-rescuing capacity of autophagy, ultimately committing both osteoblasts and endothelial cells to irreversible death.

#### 3.3.2 The “mechanistic paradox” of HIF-1α: from vascular protection to cartilage destruction

While HIF-1α is classically recognized as a protective responder to hypoxia, its role in SONFH exhibits a profound cell-specific dichotomy, which we term the “Mechanistic Paradox”. The link between ERS and this paradox is critical: severe ERS, particularly via the PERK-ATF4 axis, can directly upregulate HIF-1α transcription and stability independent of the oxygen gradient.

**FIGURE 2:**
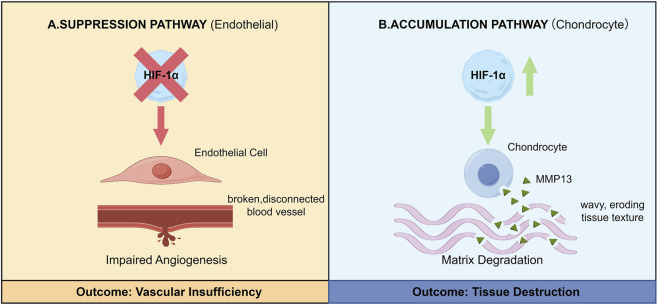
The HIF-1α Regulatory Paradox: Impaired Angiogenesis versus Matrix Degradation. **(A)** Suppressive pathway: Inhibition of HIF-1α in endothelial cells impairs angiogenesis, resulting in fragmented, discontinuous vessels and ultimate vascular insufficiency. **(B)** Accumulation pathway: Pathological accumulation of HIF-1α in articular chondrocytes activates MMP13 release, thereby attacking and degrading cartilage matrix, causing tissue destruction. (Created with Figdraw). Abbreviations: HIF-1α, hypoxia-inducible factor-1α; MMP13, matrix metallopeptidase 13; VEGF, vascular endothelial growth factor.

The physiological rationale for this divergent response is rooted in the intrinsic metabolic wiring of the distinct microenvironments. In bone marrow microvascular endothelial cells, ERS-induced HIF-1α canonically upregulates VEGF to initiate compensatory angiogenesis, serving as an “SOS” survival signal against ischemia. However, articular chondrocytes are intrinsically avascular and uniquely adapted to physiological hypoxia. Indeed, recent single-cell sequencing in degenerative cartilage models reveals that pathological ERS precipitates HIF-1α accumulation in articular chondrocytes (Sun et al., 2024), a cellular fate highly parallel to the advanced stages of SONFH. When persistent GC-induced ERS forces sustained HIF-1α accumulation in these chondrocytes, it profoundly disrupts their delicate redox homeostasis. Mechanistically, this pathological HIF-1α overload suppresses the expression of Glutathione Peroxidase 4 (GPX4) and alters iron metabolism, leading to a lethal accumulation of reactive oxygen species (ROS) and lipid peroxides. This specific biochemical cascade directly triggers chondrocyte ferroptosis (Chen B. Y. et al., 2024; Miao et al., 2022). Concurrently, this metabolic reprogramming drives the transcription of matrix-degrading enzymes, notably MMP13 (Yang et al., 2025; Mu et al., 2021). Thus, the “paradox” is elucidated: the same ERS-HIF-1α axis that promotes vascular sprouting in endothelial cells becomes a catastrophic trigger for ferroptotic death and matrix catabolism in the distinct epigenetic landscape of chondrocytes (Figure 2).

#### 3.3.3 ERS-driven lipid metabolism reprogramming

Beyond glucose metabolism dysregulation, ERS constitutes the key driver of marrow cavity “adipogenesis” (Kim et al., 2023). Glucocorticoids directly upregulate lipogenic transcription factors SREBP1 and PPARγ via the IRE1α-XBP1 axis, skewing the differentiation fate of bone marrow mesenchymal stem cells (BMSCs) toward enhanced adipogenesis at the expense of osteogenesis (Kim et al., 2023; Jiang et al., 2024). This metabolic reprogramming triggers dual pathological consequences: first, hypertrophic adipocytes mechanically compress microvessels, elevating intraosseous pressure (Jiang et al., 2024); second, excess free fatty acids generate “lipotoxicity” that further exacerbates ERS, thereby establishing a self-amplifying pathological loop of “glucocorticoid → ERS → fat accumulation → ischemia exacerbation” (Dalla Valle et al., 2019) (summarized in Table 1).

**TABLE 1:** Crosstalk between ERS and other pathological pathways in SONFH.

Interaction mode	Key mediators	Mechanistic crosstalk	Pathological outcome	Clinical implication	Ref.
ERS and Autophagy	ATG5, Caspase-3	Short-term ERS triggers protective autophagy; prolonged ERS activates ATG5-dependent apoptotic switch	Transition from cell survival to irreversible apoptosis	Need to define the specific “Therapeutic Window” for autophagy activators	Huang et al. (2022)
ERS and Angiogenesis	mTOR, HIF-1α	ERS inhibits mTOR signaling, suppressing downstream HIF-1α and VEGF translation	Impaired angiogenesis and failure of revascularization	Revascularization requires simultaneous ERS alleviation and mTOR activation	Qin et al. (2010)
ERS and Lipid Metabolism	IRE1α, XBP1, PPARγ	IRE1α-XBP1 axis directly upregulates lipogenic factors (SREBP1/PPARγ)	Fatty marrow formation; mechanical compression of vessels; lipotoxicity	Lipid-lowering agents (e.g., Statins) combined with ERS inhibitors may offer synergistic effects	Kaser et al. (2008)
ERS and Matrix Degradation	HIF-1α, MMP13	In chondrocytes, ERS stabilizes HIF-1α, which binds to the MMP13 promoter	Degradation of articular cartilage; joint collapse	HIF-1α inhibition (not activation) is required specifically in cartilage tissue	Wang et al. (2016)
ERS and Inflammation	IRE1α, JNK, NF-κB	IRE1α recruits TRAF2 to activate JNK and NF-κB pathways	Release of inflammatory cytokines (TNF-α, IL-6); exacerbation of cell death	Anti-inflammatory therapy alone is insufficient; upstream ERS blockade is essential	Liu et al. (2025)

Abbreviations: ATG5, autophagy-related 5; HIF-1α, hypoxia-inducible factor-1α; IRE1α, inositol-requiring enzyme 1α; MMP13, matrix metallopeptidase 13; mTOR, mechanistic target of rapamycin; NF-κB, nuclear factor kappa-B; PPARγ, peroxisome proliferator-activated receptor γ; SREBP1, sterol regulatory element-binding protein 1; TRAF2, TNF, receptor-associated factor 2; VEGF, vascular endothelial growth factor; XBP1, X-box binding protein 1.

ERS functions as a central hub that orchestrates complex crosstalk among autophagy, angiogenesis, lipid metabolism, matrix degradation, and inflammatory pathways in SONFH., While early adaptive responses (e.g., protective autophagy) may transiently preserve cellular homeostasis, persistent stress precipitates pathological switches (e.g., ATG5-dependent apoptosis) culminating in irreversible tissue damage. The profound heterogeneity of these interactions—exemplified by the diametrically opposed roles of HIF-1α in endothelial cells versus chondrocytes—underscores the imperative for precision therapeutic strategies tailored to specific cellular contexts.

## 4 Therapeutic strategies targeting ERS

**FIGURE 3:**
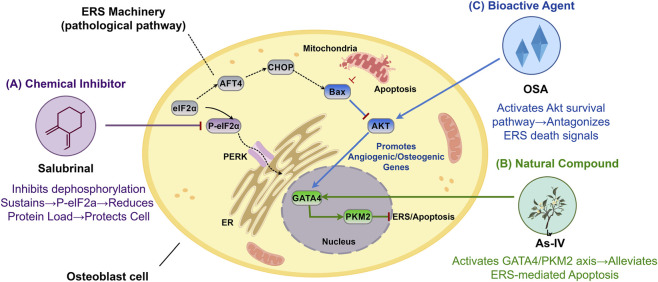
Three Therapeutic Modalities Targeting ERS Signaling Pathways in ONFH. Distinct pharmacological agents orchestrate cell fate decisions by intervening at specific nodes within the ERS network. **(A)** Chemical chaperones and small-molecule inhibitors (exemplified by Salubrinal): inhibition of eIF2α dephosphorylation reduces protein translation load and alleviates ER stress. **(B)** Natural products and TCM monomers (exemplified by astragaloside IV, As-IV): upregulation of transcription factor GATA4 promotes PKM2 expression, thereby suppressing ERS-mediated apoptosis. **(C)** Bioactive factors and gene therapy (exemplified by ortho-silicic acid, OSA): activation of Akt survival signaling antagonizes the CHOP-mitochondrial apoptotic axis and promotes cell survival (Created with Figdraw). Abbreviations: As-IV, astragaloside IV; OSA, ortho-silicic acid; P, phosphorylation; T-bar, inhibition; arrow, activation.

Given the central pathogenic role of ERS in ONFH, blocking the hyperactivated UPR signaling axis has emerged as a highly promising therapeutic target. Current intervention strategies primarily encompass three major categories: chemical chaperones and small-molecule inhibitors, natural products and traditional Chinese medicine monomers, and bioactive factors and gene therapy (Figure 3).

### 4.1 Chemical chaperones and small-molecule inhibitors

These agents function by directly modulating ER function or blocking specific kinase sites, serving as indispensable tools for validating ERS molecular mechanisms while exhibiting clinical translational potential. Salubrinal, an eIF2α dephosphorylation inhibitor, maintains eIF2α phosphorylation by specifically inhibiting its dephosphorylation, thereby reducing protein translation load and alleviating ER stress (Matsuoka and Komoike, 2015). Accumulating evidence demonstrates that Salubrinal potently suppresses glucocorticoid-induced osteoblast and osteocyte apoptosis while concomitantly promoting angiogenesis and bone healing (Matsuoka and Komoike, 2015). GSK2656157 (PERK inhibitor), a highly selective PERK antagonist, abrogates PERK autophosphorylation and downstream CHOP expression; *in vivo* validation confirms that GSK2656157 markedly attenuates vascular endothelial apoptosis, preserves femoral head microcirculation, and thereby prevents steroid-induced ONFH (Gao et al., 2020). 4-PBA (4-phenylbutyric acid), a chemical chaperone, facilitates proper protein folding and directly alleviates ER burden (He et al., 2013). Experimental findings reveal that 4-PBA significantly downregulates GRP78 and CHOP expression, reversing glucocorticoid-induced osteoblast toxicity (Kuzu et al., 2025). Levodopa/benserazide, a clinically employed anti-Parkinson agent, has been shown to ameliorate glucocorticoid-induced osteoblast apoptosis and bone loss via DRD1 activation, elevated intracellular cAMP (cyclic adenosine monophosphate) levels, and subsequent suppression of the ATF3/CHOP axis, thereby illustrating substantial potential for drug repurposing (Zheng et al., 2025).

### 4.2 Natural products and traditional Chinese medicine monomers

Traditional Chinese medicine monomers exhibit unique advantages in modulating ERS owing to their multi-target properties and low toxicity (Muehlebach and Holstein, 2025). Among these, Astragaloside IV (As-IV, effective *in vivo* at 20–40 mg/kg/day and *in vitro* at 10–50 μM) has demonstrated potent pro-angiogenic effects in SONFH models. While its ability to upregulate GATA4 expression is established, the downstream mechanism mitigating ERS-induced endothelial dysfunction warrants distinct elaboration. Specifically, GATA4 functions as a critical transcriptional survival factor; its upregulation by Astragaloside IV directly suppresses the overactivation of the PERK-CHOP apoptotic axis. Furthermore, GATA4 transactivates anti-apoptotic genes, notably Bcl-2, thereby preserving mitochondrial integrity and restoring the compromised migratory and tube-formation capacities of bone marrow microvascular endothelial cells (BMECs) under prolonged ER stress (Ouyang et al., 2023; Lorenzana-Carrillo et al., 2022). Icariin (typically administered at 20–50 mg/kg/day *in vivo* or 1–10 μM *in vitro*), a representative bioactive component of kidney-tonifying herbs, orchestrates dual therapeutic effects by concomitantly suppressing the PERK-ATF4-CHOP axis and activating Wnt/β-catenin signaling, thereby conferring both anti-apoptotic and pro-osteogenic properties (Guo et al., 2025). Puerarin (50–100 mg/kg/day *in vivo*; 10–100 μM *in vitro*) and gastrodin (50–100 mg/kg/day *in vivo*; 20–50 μM *in vitro*) have been empirically validated to markedly downregulate key ERS sensors including IRE1 and PERK, effectively mitigating oxidative stress-induced ERS damage (Li et al., 2019; Huang et al., 2025; Ye et al., 2018).

### 4.3 Bioactive factors and gene therapy

Emerging biotherapies confer unprecedented precision in therapeutic intervention. Exosome therapy: PRP-derived exosomes, enriched in VEGF and diverse growth factors, orchestrate Akt/Bad/Bcl-2 pathway activation upon cellular internalization, thereby directly antagonizing CHOP-mediated Bcl-2 downregulation—a highly promising cell-free therapeutic modality (Huang et al., 2025; Tao et al., 2017). Trace element intervention: Ortho-silicic acid (OSA) has been empirically demonstrated to mitigate endothelial ERS via Akt phosphorylation, thereby exerting dual pro-angiogenic and anti-apoptotic effects (Kuo et al., 2025; Skalny et al., 2023; Sun et al., 2024). Non-coding RNA therapy: Targeting specific miRNAs constitutes a precision strategy for ERS modulation. Specifically, *miR-182-5p* overexpression directly targets and suppresses ATF6, whereas *miR-7* concomitantly promotes osteogenic differentiation while abrogating ERS-mediated apoptosis (Kuo et al., 2025; Skalny et al., 2023; Sun et al., 2024) (Table 2).

**TABLE 2:** Summary of therapeutic agents targeting ERS in ONFH models.

Category	Agent	Target/Pathway	Mechanism of action	Experimental model	Ref.
Chemical Inhibitors	Salubrinal	eIF2α/PERK pathway	Inhibits eIF2α dephosphorylation; reduces ER load and protects osteoblasts	Rat (Surgery-induced)	Matsuoka and Komoike (2015)
	GSK2656157	PERK kinase	Highly selective PERK inhibitor; blocks CHOP expression and prevents endothelial apoptosis	Rat (Steroid-induced)	Gao et al. (2020)
	4-PBA	Chemical Chaperone	Facilitates protein folding; suppresses GRP78 and CHOP expression	Rat/Osteoblasts	He et al. (2013)
Drug Repurposing	Madopar (Levodopa)	DRD1 receptor	Activates DRD1/cAMP/PKA axis; downregulates ATF3 and CHOP.	Rat/Osteoblasts	Zheng et al. (2025)
TCM Monomers	Astragaloside IV (As-IV)	GATA4/PKM2	Upregulates GATA4 to promote PKM2 expression; inhibits ERS-mediated apoptosis	Rat (20–40 mg/kg/day)/MC3T3-E1 Cells (10–50 μM)	Ouyang et al. (2023), Lorenzana-Carrillo et al. (2022)
	Icariin	PERK and Wnt/β-catenin	Dual effect: suppresses PERK-CHOP signaling and activates Wnt pathway	Rabbit (20–50 mg/kg/day)/BMSCs (1–10 μM)	Guo et al. (2025)
	Puerarin	IRE1/CHOP	Inhibits IRE1 activation and reduces oxidative stress-induced ERS.	Rat (50–100 mg/kg/day)/Osteoblasts (10–100 μM)	Li et al. (2019)
Bioactive Agents	PRP-Exosomes	Akt/Bad/Bcl-2	Delivers growth factors to activate Akt; blocks CHOP-mediated Bcl-2 downregulation	Rat (Steroid-induced)	Liu et al. (2018), Tao et al. (2017)
	Ortho-silicic acid (OSA)	Akt/PERK	Promotes Akt phosphorylation; protects endothelial cells from ERS-induced apoptosis	Rat/HUVECs	Hahnel et al. (2020), Yuan et al. (2020)
Gene Regulation	*miR-182-5p*	ATF6	Directly targets and downregulates *ATF6* mRNA; inhibits osteoblast apoptosis	Human BMSCs	Kuo et al. (2025)

This table systematically categorizes therapeutic strategies targeting endoplasmic reticulum stress (ERS) in osteonecrosis of the femoral head (ONFH). Current interventions predominantly focus on the PERK-CHOP, apoptotic axis and the Akt survival pathway. Notably, while chemical inhibitors (e.g., Salubrinal) serve as indispensable tools for mechanistic validation, natural compounds (e.g., icariin, As-IV) and bioactive agents (e.g., PRP-exosomes) exhibit superior translational potential owing to their multi-target properties and reduced toxicity (Zhang et al., 2024). Future drug development should prioritize cell type-specific ERS, modulators integrated with bone-targeted delivery systems to overcome the “ischemic island” barrier and achieve precision joint preservation. Abbreviations: 4-PBA, 4-phenylbutyric acid; As-IV, astragaloside IV; ATF6, activating transcription factor 6; Bcl-2, B-cell lymphoma 2; BMSCs, bone marrow mesenchymal stem cells; cAMP, cyclic adenosine monophosphate; CHOP, C/EBP, homologous protein; DRD1, dopamine receptor D1; eIF2α, eukaryotic initiation factor 2α; ER, endoplasmic reticulum; ERS, endoplasmic reticulum stress; GATA4, GATA, binding protein 4; GRP78, glucose-regulated protein 78; HUVECs, human umbilical vein endothelial cells; IRE1, inositol-requiring enzyme 1; miR, microRNA; mRNA, messenger RNA; OSA, ortho-silicic acid; PERK, protein kinase R-like ER, kinase; PKA, protein kinase A; PKM2, pyruvate kinase M2; PRP, platelet-rich plasma; Wnt, Wingless/Integrated.

### 4.4 Core challenges in clinical translation

Before exploring advanced biotherapies, it is crucial to acknowledge current conservative clinical interventions. For instance, Low Molecular Weight Heparin (LMWH) and other anticoagulants are routinely employed to counteract the hypercoagulable state induced by glucocorticoids. By mitigating micro-thrombosis and reducing intraosseous hypertension, LMWH provides a fundamental degree of protection for the bone marrow microvascular endothelial network, thereby delaying the progression of early-stage SONFH (Tang et al., 2020).

#### 4.4.1 Biomechanical discrepancies between animal models and human disease progression

Current evidence overwhelmingly derives from glucocorticoid-induced quadrupedal models (rats/rabbits), wherein the femoral head is subjected to substantially inferior biomechanical loading compared with bipedal humans, with fundamentally distinct loading patterns (shear forces predominant in animals versus compressive forces in humans) (Glueck et al., 2005; Liang et al., 2025). Such disparities critically impede the faithful recapitulation of human ONFH subchondral bone collapse in late-stage disease, thereby engendering the “efficacy in animals, futility in humans” translational dilemma. Consequently, the development of biomechanically faithful ONFH animal models—exemplified by bipedal emu models or upright-induced rat models—constitutes an essential prerequisite for authentic efficacy assessment and enhanced clinical translational success.

#### 4.4.2 Physiological functions of ERS and off-target risks

ERS is not inherently pathological; rather, it constitutes a fundamental physiological mechanism whereby cells—particularly secretory-active cells such as pancreatic β-cells and plasma cells—maintain proteostasis (Gupta et al., 2025). Chronic systemic administration of broad-spectrum ERS inhibitors may disrupt UPR homeostasis in normal tissues, precipitating severe adverse effects. Specifically, the highly selective PERK inhibitor GSK2656157, despite its efficacy in osteoblast protection, has been demonstrated to induce pancreatic exocrine atrophy and aberrant glycemic metabolism (He et al., 2025). Conversely, the broad-spectrum chemical chaperone 4-PBA may elicit nonspecific cytotoxicity upon systemic high-dose application. Therefore, the development of cell type-specific ERS modulators—exemplified by agents selectively targeting osteoblasts or endothelial cells—constitutes the critical threshold for overcoming drug safety barriers.

#### 4.4.3 The “Ischemic Island” effect and delivery dilemma

**FIGURE 4:**
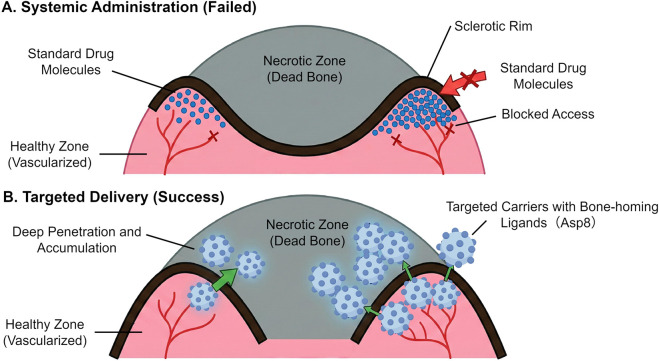
Bridging the “Ischemic Island”: Smart Delivery Strategies for Precision ONFH Therapy. **(A)** Systemic administration (free drugs): The interruption of blood supply coupled with the dense sclerotic rim constitutes an impermeable physical barrier, thereby precluding free drug access to necrotic lesions. **(B)** Bone-targeted delivery systems: Exemplified by exosomes functionalized with Asp8 peptides (a bone-homing motif), which orchestrate specific binding to hydroxyapatite within bone tissue, thereby enabling deep penetration into the necrotic core and targeted drug enrichment (Created with Figdraw). Abbreviations: Asp8, (Aspartic acid)8 peptide; ONFH, osteonecrosis of the femoral head.

The pathological essence of ONFH entails local vascular disruption. Building on radiological observations, we propose the hypothesis of an “ischemic island” model, wherein the dense sclerotic rim surrounding necrotic lesions establishes an impermeable barrier (Urra et al., 2016). Conventional systemic administration (oral or intravenous) fails to achieve therapeutic concentrations within the femoral head due to compromised microcirculation (Xiong et al., 2021). This elucidates why numerous agents demonstrating superior cellular efficacy (e.g., anticoagulants or vasodilators) exhibit minimal clinical efficacy. Consequently, future research must prioritize smart delivery systems capable of penetrating this barrier. Exemplary strategies include exosomes or nanocarriers functionalized with bone-homing peptides (e.g., Asp8) to enable site-specific drug delivery (Liang et al., 2025; Gupta et al., 2025; He et al., 2025), which may constitute a pivotal breakthrough for conquering this recalcitrant disease (Figure 4).

## 5 Discussion

### 5.1 ERS: the “central hub” orchestrating multi-dimensional pathological networks

Integrating current evidence, the role of ERS in SONFH pathogenesis transcends a mere “accompanying phenomenon”; fundamentally, it operates as a “central hub” orchestrating the connection between upstream glucocorticoid insult and downstream structural collapse (Urra et al., 2016). Previous investigations have predominantly focused on singular pathological dimensions (e.g., vascular thrombosis or lipid metabolism); however, our analysis demonstrates that ERS effectively integrates these seemingly independent processes (Xiong et al., 2021): it not only directly drives osteoblast and endothelial apoptosis via the PERK-CHOP axis but also impairs angiogenesis through mTOR pathway suppression and disrupts lipid metabolism via the IRE1-XBP1 axis. This “trinity” regulatory network underscores that SONFH essentially constitutes a systemic pathological response centered on ER homeostatic imbalance. Consequently, therapeutic strategies that attempt to circumvent ERS while targeting single branches in isolation (e.g., pure anticoagulation or lipid reduction) frequently fail to achieve satisfactory long-term clinical efficacy.

### 5.2 Signal transduction heterogeneity and “mechanistic paradoxes”

A pivotal finding of this review is the profound heterogeneity of signaling pathways within necrotic microenvironments. Classical vascular biology posits that HIF-1α functions as a protective pro-angiogenic factor; however, in SONFH articular chondrocytes, the scenario proves diametrically opposed (Li et al., 2023). Recent evidence reveals that GC-induced ERS precipitates pathological HIF-1α accumulation, which no longer confers protection but instead directly activates MMP13, thereby initiating enzymatic cartilage matrix degradation while compelling cells into hyperglycolytic metabolic traps (Zhang et al., 2019; Shan et al., 2018). This “HIF-1α paradox” fundamentally challenges the conventional dogma that “elevating HIF-1α is universally beneficial,” underscoring the imperative to incorporate cell type specificity into therapeutic design to mitigate “off-target” effects and prevent exacerbation of cartilage damage.

### 5.3 Evolution of therapeutic strategies: from single-target inhibition to systemic modulation

ERS-targeted interventions are undergoing a paradigm shift from “validation tools” to “clinical therapeutics.” Although chemical inhibitors such as Salubrinal have been instrumental in elucidating molecular mechanisms, their potential disruption of physiological UPR—particularly pancreatic β-cell secretory function—constrains translational prospects. By contrast, natural products (e.g., astragaloside IV, icariin) and bioactive carriers (e.g., PRP-exosomes) exhibit superior translational potential. These agents do not merely antagonize individual kinases but rather orchestrate pleiotropic regulatory effects—specifically suppressing pathological ERS apoptotic signals while concomitantly preserving or activating cytoprotective pathways such as Akt (Gupta et al., 2025; He et al., 2025). Furthermore, interventions targeting the “glucocorticoid-ERS-lipid accumulation” vicious loop constitute novel entry points for disrupting metabolic reprogramming.

### 5.4 Bridging the translational chasm: from molecular insights to clinical reality

Despite progressive refinement at the molecular level, it must be clearly recognized that a substantial chasm persists between basic research and clinical application. The “ischemic island” effect constitutes the core physical barrier impeding drug efficacy—compromised microcirculation within necrotic lesions precludes systemic administration from achieving therapeutic concentrations locally, thereby elucidating why numerous agents demonstrating superior *in vitro* efficacy encounter failure in clinical trials (Chen J. et al., 2024; Schlund et al., 2017). Furthermore, existing quadrupedal animal models fail to faithfully recapitulate chronic biomechanical collapse processes in humans, constituting another crucial contributor to translational failure (Xu et al., 2018; Qin et al., 2015). Future breakthroughs will hinge less on *de novo* target discovery and more on resolving delivery challenges. Specifically, the development of smart nanocarrier or exosome systems functionalized with bone-homing peptides (e.g., Asp8) to enable site-specific drug delivery represents the pivotal step toward surmounting the “ischemic barrier” and actualizing precision joint preservation (Duan et al., 2022; Thévenin et al., 2021). However, the clinical translation of these advanced delivery systems must be accompanied by rigorous safety assessments. While Asp8 peptides exhibit high affinity for hydroxyapatite, there is a potential risk of off-target accumulation in healthy epiphyseal or cortical bone. Unintended drug release in these normal skeletal sites could disrupt physiological ERS/UPR homeostasis, potentially leading to immunogenicity or unwanted cellular toxicity. To mitigate these risks, future designs should explore dual-targeting strategies or microenvironment-responsive cleavage mechanisms (e.g., ROS-responsive or acid-responsive nanocarriers) to ensure that UPR modulators are released exclusively within the ischemic and necrotic microenvironment of the femoral head (Liang et al., 2025).

### 5.5 Limitations and future directions

Nevertheless, this review is subject to certain limitations. First and foremost, current evidence overwhelmingly derives from animal models, with a paucity of validation in large-scale clinical cohorts. Second, although cellular heterogeneity of ERS signaling has been elucidated, the dynamic spatiotemporal patterns *in vivo* remain largely obscure. Looking forward, future research should prioritize: (i) the development of bone tissue-specific ERS nano-modulators; (ii) the exploration of crosstalk between ERS and other epigenetic modifications (e.g., DNA methylation, histone acetylation); and (iii) the establishment of multi-omics data-driven early warning models for ONFH to actualize genuine precision joint preservation.

## 6 Conclusion

In summary, ERS constitutes the core pathogenic driver of steroid-induced osteonecrosis of the femoral head. It orchestrates the transduction of upstream insults—specifically glucocorticoid overload and hypoxia—into downstream catastrophes encompassing cell apoptosis, vascular disruption, and metabolic dysregulation. Although the PERK-CHOP axis functions as the primary executioner of cell death, the orchestrating role of ERS within the microenvironment—particularly its profound heterogeneity across distinct cellular subpopulations—warrants equal consideration. Future breakthroughs in joint-preserving therapy imperatively necessitate grounding in profound understanding of these complex mechanisms and the seamless integration of ERS modulators with bone-targeted delivery technologies to actualize precision intervention for this recalcitrant disease.
